# Early ambulation and postoperative recovery of patients with lung cancer under thoracoscopic surgery—an observational study

**DOI:** 10.1186/s13019-023-02263-9

**Published:** 2023-04-11

**Authors:** Xiaoyun Ding, Huijun Zhang, Huahua Liu

**Affiliations:** Department of Thoracic Surgery, Huashan Hospital Fudan University, Shanghai, China

**Keywords:** Thoracoscopic surgery, Lung cancer, Early ambulation, Postoperative recovery

## Abstract

**Background:**

Enhanced recovery after surgery guidelines in China recommend early ambulation within 24 h after surgery. The aims of this audit were to investigate the early ambulation of patients with lung cancer under thoracoscopic surgery, and to explore the influence of different ambulation time on postoperative rehabilitation of patients.

**Methods:**

Using observational study method, observe and record of 226 cases under the thoracoscope surgery early ambulation of patients with lung cancer. Data collected included postoperative bowel movements, chest tube extubation time, length of hospital stay, postoperative pain and the incidence of postoperative complications.

**Results:**

The time of first ambulation was (34.18 ± 17.18) h, the duration was (8.26 ± 4.62) min, and the distance was (54.94 ± 46.06) m. The time of first postoperative defecation, the time of chest tube extubation and the length of hospital stay were significantly shortened in patients who ambulate within 24 h, and the pain score on the third day after surgery was decreased, and the incidence of postoperative complications was reduced, with statistical significance (*P* < 0.05).

**Conclusion:**

Early ambulation within 24 h after thoracoscopic surgery for lung cancer patients can promote the recovery of intestinal function, early removal of chest tube, shorten the length of hospital stay, relieve pain, reduce the incidence of complications, and facilitate the rapid recovery of patients.

Lung cancer is one of the malignant tumors with the highest morbidity and mortality in China and the world [1]. Video-assisted Thoracic Surgery (VATS) has been applied in the treatment of lung cancer due to its advantages of less surgical injury, lower incidence of postoperative complications and faster postoperative recovery [2]. According to research data, half of lobectomies performed by thoracoscopy in China in 2016 [3]. Early postoperative ambulation is one of the core measures of Enhanced Recovery After Surgery (ERAS). Early ambulation can promote the recovery of multi-system functions such as respiratory, gastrointestinal and musculoskeletal functions, and is conducive to the prevention of pulmonary infection, pressure ulcers and the formation of deep venous thrombosis of lower limbs [4]. In recent years, with the widespread use of ERAS in surgery, early postoperative ambulation is gaining more and more attention [5]. This paper aims to observe the early ambulation and postoperative rehabilitation characteristics of patients with lung cancer under thoracoscopic surgery, in order to provide reference for defining the time and specific plan of early ambulation after lung cancer surgery.

## Data and methods

### General data

Patients who underwent thoracoscopic lobectomy in the thoracic surgery ward of our hospital from January 2021 to December 2021 were selected as the study subjects. Inclusion criteria: ① Diagnosis of lung cancer by imaging examination, tracheoscopic biopsy or needle biopsy, ② The surgical method was thoracoscopic lobectomy, ③ No preoperative radiotherapy or chemotherapy was received, ④ The patients were able to take care of themselves(Barthel index ≥ 61), cooperate with others and give informed consent to this study. Exclusion criteria: ① Patients with low physical status before surgery(ASA ≥ 3), ② Patients who underwent total pneumonectomy during surgery, ③ Patients with active bleeding tendency after surgery, ④ Patients with impaired neurocognitive function or altered state of consciousness after surgery.

### Methods

#### Staff training

Unified nursing procedures and standards were developed, and 20 nurses in the thoracic surgery ward were trained by researchers and the head nurse of the ward. The training content includes theory, nursing technique operation and evaluation, observation record and so on. After unified assessment, those who pass the examination are jointly responsible for the implementation and data collection of early postoperative ambulation activities of lung cancer patients.

#### Usual postoperative care

Accordance with the Chinese Clinical Nursing Guidelines, lung cancer postoperative nursing mainly includes the following aspects: ① Ecg monitoring should be carried out according to the doctor’s advice, the vital signs of patients should be closely monitored. ② When the patient is not awake after anesthesia, take the supine position. If the blood pressure is stable after waking up, the head of the bed can be raised by 30°~45°. ③ Atomized inhalation three times a day, often assist patients to turn over, knock on the back, encourage them to cough up sputum in time. ④ If there is no gastrointestinal reaction such as nausea, vomiting and bloating 6 h after the operation, they can drink water. On the first day after the operation, they can be given a semi-liquid diet, and instructed to eat more high-vitamin and high-protein food after the operation, and drink more water to maintain intestinal patency. ⑤ The chest tube was secured and kept unobstructed. Closely observe the color, texture and quantity of drainage fluid. When the drainage fluid was significantly reduced and the drainage was less than 50ml within 24 h, the color of the drainage fluid was clear without turbidity, chest X-ray radiographs showed good lung retensification, no gas and fluid accumulation, and the patient has no dyspnea, then the extubation can be performed. ⑥ Analgesic treatment was given according to WHO guidelines for the three-step treatment of cancer pain. The time, location and degree of pain were evaluated daily after surgery. Nonsteroidal anti-inflammatory drugs were routinely used to relieve pain daily after surgery.

#### Guidance of postoperative activities

All patients adopted a unified mode of postoperative activities, and routine nursing guidance was given in accordance with the Chinese Clinical Nursing Guidelines. Encourage and assist patients to get out of bed for the first time as soon as possible according to their condition and tolerance. The patient should stand on the ground for more than 2 min for the first time to meet the requirements of getting out of bed. Specific methods: ① The patient returned to the ward after anesthesia, raised the head of the bed and the ground to 30°, and took the semi-decubed position to promote drainage and respiration. ② Active upper limb movement, including fist clenching, elbow bending, arm lifting, shoulder rotation, each movement 3 times. ③ Lower limb movement, including lower limb flexion, elevation and joint rotation, each movement 3 times. ④ Respiratory function exercise, including back tapping, deep breathing, effective cough. ⑤ Body movement in bed, including turning over and changing position in bed, relying on the strength of hands and waist and abdomen, moving hips. ⑥ Bedside sitting, that is, the hands support the legs, sit on the edge of the bed. ⑦ Bedside activities, by nurses and family members to assist standing in the bedside, sitting in the chair beside the bed, walking beside the bed. ⑧ Patients with self-activity, that is, indoor and outdoor free walking, passive to active.

#### Unexpected risk pre-control

Nurses should assess patients’ risk of falling and catheter slippage before getting out of bed, and encourage patients’ family members to participate in safety management. When the patient first gets out of bed after surgery, the nurse should inform the related precautions to prevent catheter slippage and fall. Nurse should accompany and guide the patient to move out of bed in the correct way with chest closed drainage bottle to avoid retrograde infection caused by too high drainage bottle. Nurse should remind the family member to accompany the patient every time he gets out of bed and stops the activity immediately if there is any abnormality, and inform the medical staff. Postoperative activities of patients should be gradual, in accordance with the principle of gradually increasing the amount of activity, extending the activity time and expanding the scope of activity from passive exercise to active exercise, local exercise to systemic exercise, according to the individual’s physical fitness, not too hasty. Heart rate and oxygen saturation were monitored by a mobile pulse oximeter when patients ambulate, such as the occurrence of any of the following indications that the cessation of activity: conscious palpitation, dizziness, nausea; pale face or cold sweat; SPO_2_ < 90% or heart rate reaches karvonen method [6] the target rate of sports. Target rate = (220- age - resting heart rate) × (60%∼80%) + resting heart rate.

#### Observation indicators

Early postoperative ambulation and recovery were observed. Early ambulation included the time, duration and distance of the patient’s first postoperative ambulation. Recovery included the time of first postoperative defecation, duration of chest drainage tube indwelling, postoperative hospital stay, pain and postoperative complication rate. Numerical Rating Scale (NRS) [7] was used for pain, scores 0 ~ 10 points, 0 is no pain, 10 is the patient’s pain tolerance limit, subjective score by the patient according to their own situation. According to recommendation by the European Perioperative Clinical Outcome (EPCO) Taskforce and other references, [8–10] the specific definition of each postoperative complication is presented in Table 1.

**Table 1:** Definitions of postoperative complications

Complication	Definition
Pro-longed air leak ^a^	Chest-tube drainage due to persistent air leak lasting 5 days or longer
Lung infection ^b^	Patient has received antibiotics for a suspected respiratory infection and met 1 or more of the following criteria: new or changed sputum, new or changed lung opacities, fever, white blood cell count > 12 × 10^9^/L
Atelectasis ^b^	Lung opacification with a shift of the mediastinum, hilum or hemidiaphragm toward the affected area, and compensatory over-inflation in the contralateral lung
Pleural effusion ^b^	Chest radiograph demonstrating blunting of the costophrenic angle, loss of sharp silhouette of the ipsilateral hemidiaphragm in upright position, evidence of displacement of adjacent anatomical structures or (in supine position) a hazy opacity in 1 hemithorax with preserved vascular shadows
Chylothorax ^c^	Increased thoracic drainage, and the drainage was milky after normal diet, and the chylous test of thoracic drainage was positive for Sudan III staining
Arrhythmia ^b^	Arrhythmia is defined as electrocardiograph (ECG) evidence of cardiac rhythm disturbance
Pulmonary embolism ^b^	A new blood clot or thrombus within the pulmonary arterial system

a Adapted from Society of Thoracic Surgeons and European Society of Thoracic Surgeons databases. [8]

b Adapted from recommendation by the European Perioperative Clinical Outcome (EPCO) Taskforce. [9]

c Adapted from reference of Chinses Journal of Clinical Thoracic and Cardiovascular Surgery. [10]

#### Statistical methods

SPSS 20.0 statistical software was used for data analysis. The measurement data were in line with normal distribution, expressed as mean ± standard deviation, independent sample T test was used for inter-group comparison, and counting data was used χ^2^ test or Fisher’s exact test, *P* < 0.05 was considered statistically significant.

## Results

### Postoperative patients’ early ambulation

The initial postoperative ambulation time was (34.18 ± 17.18)h, the duration was (8.26 ± 4.62)min, and the distance was (54.94 ± 46.06) m. Among them, 101 patients (44.7%) were ambulated out of bed within 24 h, 92 patients (40.7%) were ambulated out of bed within 24 to 48 h, and 33 patients (14.6%) were ambulated out of bed more than 48 h.

### Comparison of general data between the two groups

**Fig. 1:**
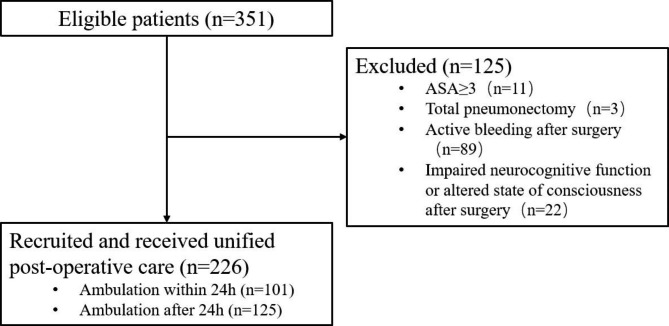
Flow of patients through the study

According to the criteria, 226 patients were included (Fig. 1). According to the time of early postoperative ambulation, patients were divided into 101 patients in the ambulation group within 24 h and 125 patients in the ambulation group after 24 h. They all underwent multiport VATS lobectomy. There were no statistically significant differences in age, gender, body mass inde, tumor site, pathological type, clinical stage and operation time between the two groups (*P* > 0.05), as shown in Table 2.

**Table 2:** Comparison of general data between the two groups

Clinical features	Ambulation within 24 h (n = 101)	Ambulation after 24 h (n = 125)	Statistical quantity	*P* value
Age, years	54.12 ± 12.58	57.26 ± 12.62	1.861	0.064^*^
Gender, n			0.825	0.364
male	48	67		
female	53	58		
BMI, kg/m^2^	23.29 ± 2.80	23.79 ± 3.12	1.259	0.209^*^
Tumor site, n			7.608	0.107
Left upper lung	24	33		
Left lower lung	7	20		
Right upper lung	34	33		
Right middle lung	13	8		
Right lower lung	23	31		
Pathological type, n			0.479	0.759^**^
Squamous cell carcinoma	7	11		
Adenocarcinoma	91	109		
Other	3	5		
TNM stage, n			0.671	0.715
I	77	98		
II	19	19		
III and above	5	8		
Operation time, min	114.55 ± 44.04	119.41 ± 43.00	0.835	0.405^*^

Note: * represents t test, ** represents Fisher’s exact test, and the rest is χ^2^Inspection

### Comparison of the time of first postoperative defecation, chest tube extubation and the length of hospital stay in the two groups

The time of first postoperative defecation, chest tube extubation and the length of hospital stay in the ambulation group within 24 h were significantly lower than those ambulation group after 24 h, with statistically significant differences (*P* < 0.05), as shown in Table 3.

**Table 3:** Comparison of the time of first postoperative defecation, chest tube extubation and the length of hospital stay between the two groups

	Ambulation within 24 h (n = 101)	Ambulation after 24 h (n = 125)	*t* value	*P* value
The time of first defecation, d	2.73 ± 1.14	3.38 ± 1.34	3.863	< 0.01
The time of chest tube extubation, h	81.36 ± 60.13	111.69 ± 62.42	3.692	< 0.01
The length of hospital stay, d	4.32 ± 2.66	5.96 ± 2.49	4.780	< 0.01

### Comparison of postoperative pain scores between the two groups

In the first and second day after surgery, there was no significant difference in pain scores between the two groups (*P* > 0.05). In the third day after surgery, the pain scores of the group that ambulate within 24 h was lower than that of the group ambulate after 24 h, the difference was statistically significant (*P* < 0.05), as shown in Table 4.

**Table 4:** Comparison of postoperative pain scores between the two groups

Pain score	Ambulation within 24 h (n = 101)	Ambulation after 24 h (n = 125)	*t* value	*P* value
Postoperative day 1	2.54 ± 1.26	2.42 ± 1.27	0.761	0.448
Postoperative day 2	2.36 ± 1.13	2.26 ± 1.06	0.635	0.526
Postoperative day 3	1.31 ± 0.96	1.95 ± 0.51	6.501	< 0.01

### Comparison of postoperative complications between the two groups

The incidence of postoperative complications in the ambulation group within 24 h was lower than that ambulation group after 24 h, and the difference was statistically significant (*P* < 0.05), as shown in Table 5.

**Table 5:** Comparison of postoperative complications between the two groups

Postoperative complications, n(%)	Ambulation within 24 h (n = 101)	Ambulation after 24 h (n = 125)	χ^2^ value	*P* value
Pro-longed air leak	7 (6.9)	21 (16.8)		
Lung infection	0 (0.0)	1 (0.8)		
Atelectasis	0 (0.0)	2 (1.6)		
Pleural effusion	1 (1.0)	2 (1.6)		
Chylothorax	1 (1.0)	1 (0.8)		
Arrhythmia	1 (1.0)	1 (0.8)		
Pulmonary embolism	1 (1.0)	0 (0.0)		
Total	11 (10.9)	28 (22.4)	5.182	0.023

## Discussion

### Implementation of early ambulation in patients with lung cancer under thoracoscopic surgery

At present, there is no unified definition of early ambulation after thoracoscopic surgery. Among the foreign concepts of accelerated rehabilitation surgery, Gatt etc. [11] defined early postoperative ambulation was ambulation on the day of surgery and the distance of walking corridor on the first day after surgery. Ramfrez etc. [12] defined early postoperative ambulation was ambulation on the first postoperative day and sedentary rest for at least 6 h. Other researchers started early ambulation after lung resection in patients in advance to postoperative 4 h, the chest tube did not appeared a major problem, the patient did not fall, and the heart rate and the change of pain when walking with the control group, there was no difference between confirmed lung resection of lung cancer patients with postoperative 4 h safety of ambulation [13]. Chinese people’s medical publishing house published the fifth edition of the surgical nursing, expounds the principles of early ambulation after surgery, “the majority of patients within 24 h ~ 48 h after try out of bed, in a stable condition after activities encourage patients early bed, try to get up in the short term, unless there are special requirements, encourage and assist patients in bed to take a deep breath, to turn over, limbs activity” [14]. In the expert consensus and management guidelines of the 2018 edition of accelerated rehabilitation surgery in China, it is recommended that Fowler’s position or right amount of bed activities can be carried out after being awake after surgery, and early ambulation out of bed can be started on the first day after surgery, and daily activity goals can be established to increase the amount of activity day by day [4]. In this study, the early ambulation time of patients with lung cancer under thoracoscopic surgery was (34.18 ± 17.18) h, and there were still 33 patients who did not start early ambulation until more than 48 h after surgery. These patients are deeply rooted in the Chinese traditional concept of bed rest after surgery, but existing studies have shown that bed rest after surgery has many harms, such as decreased insulin sensitivity, atelectasis, decreased exercise ability, muscle atrophy, bone loss, thrombotic diseases, microvascular dysfunction, stress injury etc. [15]. Therefore, nurses still need to strengthen the health education of patients, change the concept of patients, so as to improve the compliance of early postoperative ambulation activities.

### Early ambulation postoperatively in patients with lung cancer promote postoperative lung and gastrointestinal function recovery

Chest tube in the lung cancer patients is placed in order to drainage the pleural cavity effusion and pneumatosis, rebuild the pleural cavity negative pressure, promote lung recruitment, as well as to help medical staff to observe the chest cavity with and without active bleeding, pulmonary air leakage etc. To promote the recovery of lung function and reduce the incidence of lung infection, its management is one of the important links of postoperative nursing for lung cancer patients [16]. Early postoperative ambulation can promote the early discharge of pleural effusion in lung cancer patients, facilitate the early recovery of lung, promote the early removal of thoracic drainage tube, and shorten the length of hospital stay [17]. In terms of gastrointestinal function recovery, early postoperative out of bed activity can promote local and systemic blood circulation, while the change of position can also cause gastrointestinal reflex, thus promoting gastrointestinal peristalsis, improving gastrointestinal flatulence, shortening the time of anal exhaust defecation, and speeding up gastrointestinal function recovery [18]. The results of this study showed that the time of first postoperative defecation, the time of chest tube extubation and the length of hospital in the ambulation group within 24 h were significantly lower than those ambulation group after 24 h, with statistically significant differences (*P* < 0.05), indicating that early ambulation within 24 h postoperative in lung cancer patients is beneficial to promote the recovery of lung recovery and intestinal function, shorten postoperative chest tube indwelling time and postoperative hospitalization time, effectively promote the rapid postoperative recovery of patients.

### Early ambulation postoperatively in patients with lung cancer is beneficial to relieve postoperative pain

A survey of postoperative pain experience shows that about 80% of patients said they had acute pain after surgery [19]. Postoperative pain is an independent risk factor for complications after lobectomy [20]. The pain after lung cancer surgery will make patients have fear of cough, which is not conducive to sputum discharge, easy to cause lung infection, atelectasis and other pulmonary complications, affecting postoperative quality of life [21]. Postoperative pain will also lead to neuroticism, peripheral and central release media, resulting in tachycardia, myocardial ischemia and poor wound healing and other adverse reactions, delay the recovery of patients [22]. Feng Rui etc. [23] indicates that the width of the rib gap are the main factors influencing the lung cancer patients with postoperative pain, postoperative, except for conventional analgesic interventions in patients with early ambulation by guiding patients to take a deep breath and effective cough, such as tensile side body movement, can open the width of the rib clearance patients, so as to reduce the chest drainage tube and the rib clearance friction produced by the pain. From the perspective of psychological factors, postoperative activities are beneficial to divert patients’ attention and increase recovery confidence. With the reduction of patients’ negative emotions, happiness is improved, which is more conducive to pain control [24]. In this study, many patients were affected by the traditional concept that stay in bed is necessary after surgery, they think early activities will cause severe pain. However, the results of this study showed that there was no statistically significant difference in pain scores between the two groups on the 1st and 2nd day after surgery (*P* > 0.05), and the pain scores of patients in the ambulation group within 24 h on the 3rd day after surgery were lower than those ambulation group after 24 h, the difference was statistically significant (*P* < 0.05). It can be seen that early postoperative ambulation of lung cancer patients not only does not aggravate the pain, but is beneficial to the relief of postoperative pain in the later stage.

### Early ambulation postoperatively in patients with lung cancer is beneficial to reduce postoperative complications

Due to surgical trauma, a large amount of gas and fluid accumulation in the pleural cavity, combined with the effects of intraoperative anesthesia, postoperative pain, fear and other factors, the cough ability of lung cancer patients after surgery is limited, resulting in the occurrence of lung infection and other complications [25]. It has been reported that the complication rate of patients undergoing thoracoscopic lung cancer surgery fluctuates from 2–40% [26], and an Chinese study reported a complication rate of 15% after thoracoscopy [27]. Grade etc. [28] believe that early ambulation is the key measure to prevent postoperative pulmonary complications, which is beneficial to patients’ postoperative recovery. Through early postoperative active guidance to patients with lung cancer, step by step from the time, frequency and methods to carry out activities, prevent the patient from the blind activities adverse reactions, and respiratory function exercise by enhancing well and the power of the auxiliary well improved lung function, and promoted the airway secretion eduction, thus reduce pulmonary complications [29, 30]. In addition, appropriate exercise intervention can also improve the overall physiological muscle function of patients and reduce the postoperative sequelae of skeletal muscle [31]. In this study, the incidence of postoperative complications of patients in the ambulation group within 24 h was lower than those ambulation group after 24 h, the difference was statistically significant (*P* < 0.05), indicating that ambulation within 24 h after surgery is beneficial to reduce postoperative complications in lung cancer patients. However, in this study, there was still one patient with postoperative pulmonary embolism in the ambulation group within 24 h, and no pulmonary embolism occurred in the ambulation group after 24 h, which may be related to the patient’s previous medical history, gynecological malignant tumor and thyroid tumor surgery.

## Summary

Early ambulation can promote the recovery of various physiological abilities of lung and body after lung cancer patients, reduce postoperative pain, reduce postoperative complications, so as to achieve the purpose of rapid recover. So early ambulation has high clinical value. However, at present, there are no clear standards or guidelines for the best time, intensity and activity type of early ambulation, and there are many and complex risk factors for early ambulation. How to accurately and effectively implement early ambulation, an important postoperative nursing measure, is worthy of further study in the future.

## Data Availability

Not applicable.

## Declarations

### Open access

Not open access.
